# Impact of COVID-19 on Infertility Treatments: Not Even a Global Pandemic Was Strong Enough to Hamper Successful Pregnancies

**DOI:** 10.3390/life12010006

**Published:** 2021-12-21

**Authors:** Cristina Rodríguez-Varela, Giulia Mariani, Pilar Dolz, Juan Antonio García-Velasco, Vicente Serra, Antonio Pellicer, Elena Labarta

**Affiliations:** 1 IVI Foundation—IIS La Fe, Avenida Fernando Abril Martorell, Building 106 A, 7th Floor, 46026 Valencia, Spain; elena.labarta@ivirma.com; 2 IVI RMA Rome, Largo Ildebrando Pizzetti, 1, 00197 Rome, Italy; giulia.mariani@ivirma.com (G.M.); apellicer@ivirma.com (A.P.); 3 IVI RMA Valencia, Plaza Policía Local 3, 46015 Valencia, Spain; pilar.dolz@ivirma.com (P.D.); vicente.serra@ivirma.com (V.S.); 4 IVI RMA Madrid, Avenida del Talgo 68, 28023 Madrid, Spain; juan.garcia.velasco@ivirma.com

**Keywords:** COVID-19, coronavirus, pregnancy, pandemic, infertility treatments

## Abstract

The COVID-19 global pandemic has meant a sanitary and social threat at every level and it was not any different for the assisted reproduction industry. This retrospective two-arm study aims to describe its impact on infertility treatments performed in our clinics (IVI Spain, Rome, and Lisbon) regarding: (1) assessment of COVID-19 impact in the amount, type, and success of infertility treatments performed during 2020 compared to 2019; and (2) description of the psychological status of women who got pregnant during the first months of the pandemic and its correlation with their final pregnancy outcome. On the one hand, this pandemic has led to a significant reduction in the total number of treatments performed, even though the proportion of the different types was almost unaltered. Additionally, its impact on pregnancy rates was not clinically relevant. On the other hand, the psychological status of pregnant women did not seem to affect their final pregnancy outcome. These results suggest that, even in the event of a negatively affected psychological status in our study population, it was not translated into an impaired pregnancy outcome. Hence, the COVID-19 global pandemic, although devastating, might not have exerted a clinically relevant negative impact on the overall pregnancy outcome in our clinics.

## 1. Introduction

COVID-19 disease, secondary to SARS-CoV-2 infection, has caused a global pandemic since it was reported at the end of 2019 in China [1]. Its high contagiousness has forced us to stop our lives and even our jobs completely for a few months and, in a more relaxed manner, with fewer restrictions afterwards. This impasse has also affected assisted reproduction techniques (ART) as the number of treatments was drastically reduced due to the unknown effect of COVID-19 on pregnancy and obstetric outcomes [2].

In Europe, the first confirmed COVID-19 case was detected in France at the end of January [3]. From this moment on, ART treatments began to decline in Spanish clinics as a significant proportion of our patients are of foreign origin. Soon after, the first COVID-19 case in Spain was detected in La Gomera [4]. ART continued with this huge decline until the closure of all infertility clinics at the end of March, right after several professional societies around the world recommended to suspend infertility services [2]. Clinics reopened in mid-May and, since then, the normal cycle rhythm has been gradually recovering.

Besides the huge impact on the ART industry, this pandemic is also a sanitary threat and thus it may affect pregnant women and their offspring. At the beginning of this pandemic, there were few data regarding the virus effects on pregnancy and thus the increased fear in starting any infertility treatment. Over the course of 2020 and 2021, more data on pregnant women with and without the infection became available [5,6]. It seems that the COVID-19 pandemic did not compromise ART early pregnancy outcomes in comparison to a pre-COVID time frame [6], even though obstetric and perinatal complications were higher in COVID-positive pregnant women, in particular if they were symptomatic [5]. However, apart from a physical threat, this pandemic has also exerted a great impact on the population’s psychological health. The lockdown period, fear of the unknown, uncertainty about when this situation will end, and worry about sick family members or friends are only examples of all the mental threats the whole population is currently experiencing.

Nowadays, it is believed that the mental health and psychological status of patients influences their ART cycle outcome, as well as pregnancy evolution in those who get pregnant, exerting its effect through the hypothalamus–pituitary–adrenal axis and sympathetic nervous system. Higher levels of stress in women undergoing ART treatments, measured by different biomarkers (cortisol, α-amylase, and norepinephrine) and standardized psychological questionnaires, have been related to lower pregnancy and live birth rates, as well as longer time to pregnancy [7,8,9]. Given the high psychological load of this pandemic, we wanted to evaluate its impact on our pregnant patients’ mental health, how it progressed throughout pregnancy, and if this impact was somehow related to their final pregnancy outcome.

In addition, given the huge drop in the number of ART treatments performed during 2020, we wanted to compare our overall pregnancy rates to the ones registered in the previous year. In this manner, we would be able to assess if the COVID-19 pandemic only negatively affected the number of treatments or if it has also reduced the chances of achieving a successful pregnancy.

## 2. Materials and Methods

### 2.1. Design and Setting

This is a retrospective, multicentric, and double-arm study conducted in all IVI clinics in Spain, along with IVI Lisbon and IVI Rome. 

The first arm includes data from all treatments performed from 12 February onwards in 2019 and 2020. 

The election of 12 February as the start date is based on the fact that from that date onwards, all pregnancy tests were done while being more conscious of the critical situation in Italy, which led to harder sanitary and political actions regarding the pandemic.

The second arm includes data from pregnant patients whose first trimester coincided with the onset of the pandemic (February and March 2020) and who had responded to at least one of the three pregnancy follow-up surveys. 

### 2.2. Study Procedures

First arm:

Descriptive analysis of all infertility treatments (amount, type, success rates, etc.) performed between 12 February and 31 December 2020. Data from the same time period in 2019 has been taken as a control group in the comparative analysis.

Second arm:

Descriptive analysis of the psychological status of pregnant women whose first trimester coincided with the onset of the COVID-19 pandemic in Spain and the assessment of its impact in their final pregnancy outcome. This psychological status has been evaluated while taking into account several answers from their pregnancy follow-up surveys.

As part of our routine clinical practice, pregnant patients receive several phone calls during their pregnancy in order to ask about its evolution. Given the sanitary situation and the establishment of distance working, we substituted these phone calls for surveys sent over e-mail. 

Data from all these surveys was exported and analyzed, as well as the final pregnancy outcome of all those patients who had answered to at least one of the surveys. The impact of the pandemic on the psychological status of pregnant women has been evaluated, as well as the subsequent impact of this psychological status on final pregnancy outcome. In addition, the evolution of their anxiety status throughout pregnancy and pandemic has been also assessed.

Anxiety levels were assessed using an adjusted Hamilton Scale, in which one of the 14 questions could not be evaluated as the evaluation did not occur in person [10]. The choice of this scale was due to its standardization and worldwide validation in the absence of any salivary or serum biomarker measurement. Patients were then divided into three main categories: mild, mild to moderate, and moderate to severe anxiety. This scale was complemented with many other questions, in which variables could have directly or indirectly affected patients’ anxiety levels. These questions include: levels of concern regarding the pandemic, diet, exercise, family members affected by COVID-19, etc.

### 2.3. Statistical Analysis 

A statistical analysis was performed using IBM SPSS 15.0.1 Statistics for Windows (SPSS Inc. Chicago, IL, USA). Quantitative variables are presented with the mean (95% confidence interval) and categorical variables with n (percentage). ANOVA and ꭓ^2^ tests were employed for comparisons between variables. A *p*-value < 0.05 was defined as statistically significant. 

## 3. Results

### 3.1. Descriptive Analysis of Infertility Treatments in 2020 versus 2019

#### 3.1.1. Types and Quantity of Infertility Treatments Performed

In 2019, a total of 23,078 embryo transfers or IUIs were performed between IVI clinics in Spain, IVI Lisbon, and IVI Rome, in contrast to the 19,261 performed in 2020. Cycle and demographic patients’ characteristics are summarized in Table 1.

Table 2 presents a detailed descriptive analysis of these cycles regarding the type of treatment.

**Figure 1:**
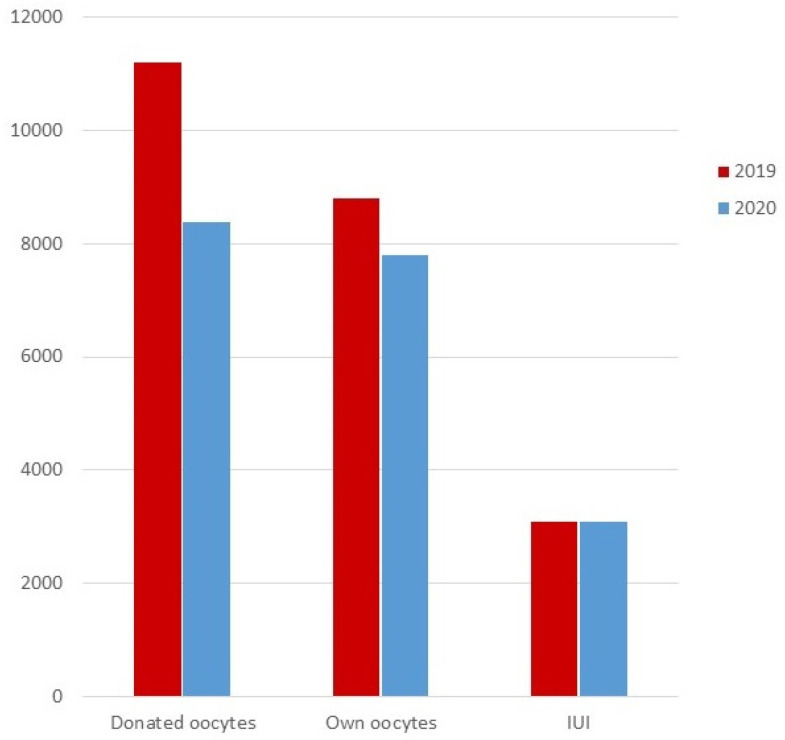
Number of procedures regarding the three main types of treatment (own oocytes, donated oocytes, and intrauterine insemination) in 2019 (red) and 2020 (blue). IUI = intrauterine insemination.

Figure 1 represents the comparison in the number of cycles performed in 2019 vs. 2020 regarding the three main categories of type of treatment (IUI and embryo transfers from own and donated oocytes). 

#### 3.1.2. Timeline of the Amount of Infertility Treatments Performed in 2020 versus 2019

**Figure 2:**
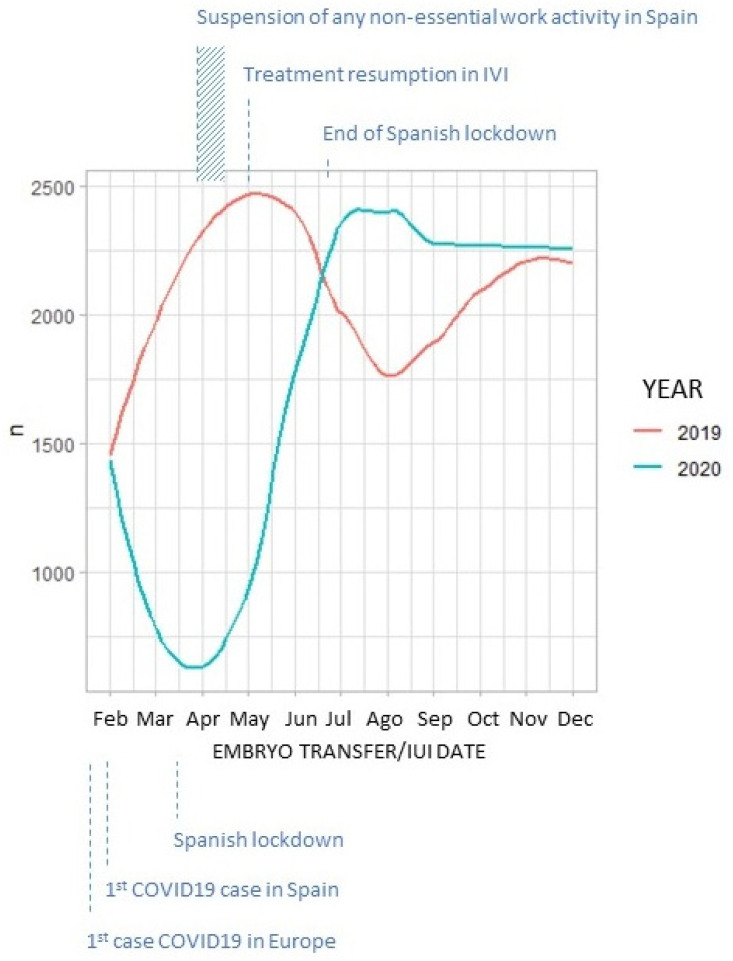
Representation of the total number of embryo transfers/intrauterine inseminations performed between February and December in 2019 (red) and 2020 (blue).

In 2020, the total number of treatments performed in our clinics per month followed a completely opposite trend to that registered in 2019. This trend corresponds to the episodes that took place in Spain during this period (Figure 2).

#### 3.1.3. Infertility Treatments Conducted in Spain Regarding Patients’ Country of Residence

**Figure 3:**
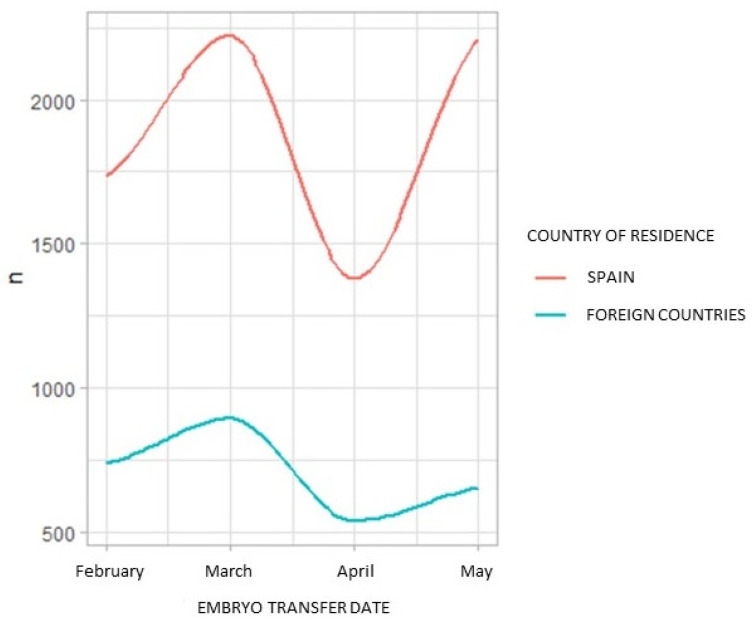
Number of procedures performed during the first three months of the pandemic in Spanish clinics, in patients of foreign (blue) and Spanish (red) origin.

The first COVID-19 cases in Europe appeared in other countries before arriving in Spain. Figure 3 represents the decline in the number of infertility treatments in our Spanish clinics during the first three months of the pandemic, both in patients of foreign (blue) and Spanish (red) origin.

#### 3.1.4. Gestational Results Registered in 2020 versus 2019

Mean pregnancy rates in 2019 and 2020, as well as their comparison, are presented in Table 3 regarding the type of treatment (IUI and IVF/ICSI with own oocytes and IVF/ICSI with donated oocytes).

### 3.2. Impact of COVID-19 in Gestational Results and Psychological Status of Women Whose First Trimester of Pregnancy Corresponded with the Onset of the Pandemic in Spain

In 2020, we gathered data from a total of 874 patients who got pregnant during the first three months of the pandemic and who answered to at least one of the surveys. In the first trimester, there were 439 answers from 1171 surveys sent (37.5%). In the second trimester, there were 614 answers from 2126 surveys sent (28.9%). However, only 341 from these 614 answers are from women whose first trimester coincides with the very onset of the pandemic (February and March, 2020). Finally, there were 140 answers from 719 surveys sent (21.3%) during the post-partum period. Some patients have answered more than one survey. Only 71 patients answered all three surveys. 

A descriptive analysis of the final pregnancy outcome of these 874 patients is presented in Table 4. Unfortunately, we lack information on the final pregnancy outcome of 135 ongoing pregnancies beyond the 12th week. 

A descriptive analysis of the patients’ answers to our follow-up surveys is presented in Table 5. 

In order to ease the comparative analysis between the answers to the surveys and the final pregnancy outcome, this later variable was encompassed in two main categories: live birth (689/739) and miscarriage or gestational loss (50/739). Ongoing pregnancies beyond the 12th week lost to follow-up (n = 135) were withdrawn from the comparative analysis.

Regarding the Hamilton scale results, there was not any significant impact on pregnancy outcome on the first (*p* = 0.598) and second trimester (*p* = 0.745). In the post-partum survey, however, almost only pregnancies with live birth answered to these anxiety scale questions (107 answers in the live birth group vs. 1 in the miscarriage group; 30 answers in the ongoing pregnancy group, which were not analyzed due to the lack of final pregnancy outcome results). Therefore, the correlation between the Hamilton scale results assessed during the post-partum period and the final pregnancy outcome could not be analyzed.

A similar situation occurred with the data regarding levels of concern, levels of exercise, diet, if someone related died for COVID-19, or their COVID-19 symptoms/test result in the first and second trimester. In line with the general results (Table 5), the majority of pregnant women in both the live birth and miscarriage groups had high levels of concern, their physical exercise had been reduced, their diet was unchanged, no one close died from COVID-19, and they lacked any COVID-19 symptoms (*p* = 0.000). Hence, due to the high homogeneity in the answers between both pregnancy outcome groups and added to the large difference in sample size between the miscarriage (n = 24 answers) and live birth groups (n = 347 answers), the potential impact of these variables on final pregnancy outcome could not be correctly evaluated. 

Finally, a descriptive analysis of the six pregnant women who tested positive for COVID-19 (one in the survey of the first trimester and five in the survey of the second trimester) showed that all of them reported mild anxiety levels as measured by the Hamilton scale and ended in live birth. In contrast, levels of concern regarding the pandemic were moderate and high in two and four of them, respectively. 

## 4. Discussion

The onset of the COVID-19 global pandemic has forced the whole world to stop and infertility treatments were not any different. In the current study, we wanted to show an overall perspective on the impact of this pandemic in the ART sector, as well as in the reproductive outcome of those patients who got pregnant in our clinics during its most crucial months. 

Cycle and demographic characteristics of our patients in 2020 were comparable to the ones in 2019. In 2020, however, women undergoing ovarian stimulation protocols needed more days of stimulation and thus received higher FSH doses. These differences, although statistically significant, were not clinically relevant. In addition, estradiol levels in the triggering day were significantly lower in 2020, suggesting that these women had a slower response to ovarian stimulation and thus the higher days of FSH exposure (Table 1).

In general, the total number of infertility treatments performed in 2020 suffered a huge decline in comparison to the ones performed in 2019 regardless of the type of treatment. Indeed, the proportion of the different types of treatments remained practically the same in both years (Table 2 and Figure 1). Fresh embryo transfers with donated oocytes were slightly reduced in 2020 probably due to the added difficulty of synchronizing donor and patient. 

Additionally, this decline followed a trend that corresponds to the episodes that took place in Spain during this period. Hence, there was an initial huge drop in the number of procedures during the first months of the pandemic. Once infertility treatments resumed in the spring, however, the number of procedures exceeded the ones registered during the same period in 2019, as a substantial drop in the number of treatments performed is usually experienced due to the holiday season (Figure 2). 

The specific situation of foreign patients coming to IVI Spain in order to undergo an infertility treatment is of particular interest. COVID-19 cases began to appear in other European countries before they did in Spain, thus patients coming from abroad also began to decline with the onset of the pandemic. Furthermore, the closure of country borders totally stopped reproductive tourism in our country (Figure 3).

Despite the evident negative effect on the number of infertility procedures, the pandemic and its associated sanitary crisis did not seem to affect pregnancy success rates. Differences in pregnancy and clinical pregnancy, although significant due to the high sample size, were not clinically relevant. In contrast, ongoing pregnancy (defined as ongoing pregnancies with a gestational sac that did not suffer any miscarriage) and live birth rates, as well as biochemical and clinical miscarriages, did not show any statistically significant difference between 2019 and 2020 (Table 3). Hence, despite the lower total number of treatments, those couples who decided to undergo an infertility treatment in 2020 had the same chances of success as the previous year.

Along with successful pregnancy rates, answers to our follow-up surveys showed that these patients were not suffering from high levels of stress. This situation did not vary as pregnancy evolved, even though they did acknowledge high levels of concern regarding the pandemic during the first and second trimester (Table 5).

In general, patients’ lifestyle did not change much as a result of this sanitary crisis. Their physical exercise was reduced, whereas their diet remained unchanged. Our study population did not suffer the death of someone close due to COVID-19 and they did not have any symptoms themselves (Table 5).

However, it was not possible to evaluate the impact of these answers on final pregnancy outcome due to the far-from-heterogeneous distribution of these variables, as the majority of patients showed a clear trend towards one specific common answer. In addition, almost only good prognosis pregnancies are represented in our study population (Table 4). It is understandable that pregnancy losses and pregnancies with early worse prognosis were not in a good place to fill these surveys. Hence, our results have an important bias and they almost describe those pregnancies with good final outcomes.

It is important to take into account that patients with any kind of miscarriage or arrested pregnancy may not have answered our surveys. Most patients with a biochemical miscarriage might have received the questionnaire from the first trimester after their loss. In addition, these patients have not answered the second and third surveys, as they were not pregnant anymore. This explains the reduced sample size regarding this pregnancy outcome. A similar situation occurred with clinical miscarriages and arrested pregnancies.

Additionally, any attempt to assess the evolution in time of psychological status and levels of concern in those patients who answered all of the surveys (n = 71) failed. As expected, these patients correspond to those with live birth or ongoing pregnancies, thus good prognosis patients and again a bias was observed. 

Finally, the sub-analysis performed in the sub-population of COVID-19-positive pregnant women, although of very low sample size (n = 6), does not suggest any impact of the disease in these patients’ psychological status and final pregnancy outcome. In this sub-population, high levels of concern were not translated into an increased anxiety or impaired final pregnancy outcome.

In any case, results from the first arm clearly show no significant clinical difference in pregnancy outcome between two consecutive years with and without a huge source of stress, as it is the case of the COVID-19 pandemic. Therefore, regardless of the bias described in the second arm of the study, the overall pregnancy outcome in infertility treatments was not affected by the COVID-19 pandemic and stress generated by this situation.

## 5. Conclusions

The course of the pandemic over time is clearly reflected in the amount of infertility treatments performed, as well as in the re-productive tourism in our country. However, success rates and the proportion of the different types of treatments were quite similar to the ones registered in the previous year. Additionally, we have provided a brief description of the psychological status of patients who got pregnant during the first months of this pandemic. Results from both study arms suggest that the COVID-19 global pandemic, although devastating, does not seem to have exerted a clinically relevant negative impact on the overall pregnancy outcome in our clinics. Despite this, the main limitation of the current study is the bias resulting from the follow-up surveys answered, which might have been returned mostly by patients with good prognosis pregnancies. In contrast, pregnancy data do constitute a source of objective information. Thus, in the event of a potentially negatively affected psychological status not detected by our surveys, it was not translated into an impaired pregnancy outcome.

## Figures and Tables

**Table 1:** Cycle and demographic characteristics of our patients in 2019 and 2020.

Variable	2019			2020			*p*-Value ^a^
	n	Mean	95% CI	n	Mean	95% CI	
Age	23,078	38.9	38.8–39.0	19,261	38.6	38.6–38.7	**0.000**
Donor age	11,251	25.4	25.4–25.5	8443	25.5	25.4–25.6	0.691
BMI	13,456	24.0	23.4–24.5	12,726	23.4	23.3–23.5	0.077
Days of stimulation	18,773	15.9	15.9–15.9	16,424	16.1	16.1–16.1	**0.000**
E2 day of trigger	2980	836.7	805.7–867.8	2979	789.1	758.5–819.8	**0.032**
P4 day of trigger	3266	0.6	0.4–0.9	3091	0.4	0.4–0.5	0.192
AMH	9235	6.1	5.8–6.3	9539	6.3	6.1–6.5	0.240
AFC right ovary	11,789	6.2	4.6–7.9	11,617	4.9	4.8–5.0	0.112
AFC left ovary	11,775	6.1	4.4–7.7	11,597	4.8	4.7–5.0	0.148
FSH total dose	3631	970.2	943.7–996.8	3432	926.6	899.8–953.4	**0.023**
hMG total dose	2200	979.9	953.7–1006.1	1995	1003.6	974.1–1033.1	0.237

^a^ ANOVA. n = number of cases analyzed; 95% CI = 95% confidence interval; BMI = body mass index; E2 = estradiol; P4 = progesterone; AMH = anti-mullerian hormone; AFC = antral follicle count; FSH = follicle-stimulating hormone; and hMG = human menopausal gonadotropin.

**Table 2:** Number of procedures regarding the type of treatment in 2019 and 2020.

Treatment	2019	2020	*p* Value ^a^
	n = 23,078	n = 19,261	
FET Own Oocytes	3675 (15.9%)	3240 (16.7%)	**0.013**
FET own oocytes with PGT	2899 (12.6%)	2621 (13.6%)	**0.001**
Fresh embryo transfer own oocytes	2229 (9.7%)	1926 (10.0%)	0.240
Homologous/Donor IUI	3075 (13.3%)	3086 (16.0%)	**0.000**
FET donated oocytes	6309 (27.3%)	5062 (26.3%)	**0.015**
FET donated oocytes with PGT	440 (1.9%)	371 (1.9%)	0.884
Fresh embryo transfer donated oocytes	4451 (19.3%)	2955 (15.3%)	**0.000**

FET = frozen embryo transfer; PGT = preimplantational genetic test; and IUI = intrauterine insemination. ^a^ ꭓ^2^ test.

**Table 3:** Gestational results in 2019 versus 2020 regarding the type of treatment (IUI and IVF/ICSI with own oocytes and IVF/ICSI with donated oocytes).

	Treatment	2019	2020	*p*-Value
**Pregnancy**	Homologous/Donor IUI	696/3075 (**22.6%**)	627/3086 (**20.3%**)	**0.015 ^a^**
	IVF/ICSI own oocytes	4979/8752 (**53.9%**)	4247/7732 (**54.9%**)	**0.006 ^a^**
	IVF/ICSI donated oocytes	6607/11,251 (58.7%)	4961/8443 (58.8%)	0.486 ^a^
**Clinical** **Pregnancy**	Homologous/Donor IUI	597/3075 (19.4%)	555/3086 (18.0%)	0.080 ^a^
	IVF/ICSI own oocytes	4323/8752 (**49.4%**)	3713/7732 (**48.1%**)	**0.041 ^a^**
	IVF/ICSI donated oocytes	5782/11,251 (51.4%)	4369/8443 (51.8%)	0.315 ^a^
**Ongoing** Pregnancy *	Homologous/Donor IUI	488/3075 (15.9%)	457/3086 (14.8%)	0.131 ^a^
	IVF/ICSI own oocytes	3531/8752 (40.4%)	3045/7732 (39.4%)	0.107 ^a^
	IVF/ICSI donated oocytes	4726/11,251 (42.0%)	3607/8443 (42.7%)	0.160 ^a^
**Live Birth**	Homologous/Donor IUI	477/3075 (15.5%)	446/3086 (14.5%)	0.129 ^a^
	IVF/ICSI own oocytes	3480/8752 (39.8%)	3003/7732 (38.8%)	0.116 ^a^
	IVF/ICSI donated oocytes	4671/11,251 (41.5%)	3566/8443 (42.2%)	0.159 ^a^
**Biochemical** **Miscarriage**	Homologous/Donor IUI	100/696 (14.4%)	74/627 (11.8%)	0.097 ^a^
	IVF/ICSI own oocytes	661/4979 (13.3%)	537/4248 (12.6%)	0.191 ^a^
	IVF/ICSI donated oocytes	829/6607 (12.6%)	597/4961 (12.0%)	0.211 ^a^
**Clinical** **Miscarriage**	Homologous/Donor IUI	109/597 (18.3%)	98/555 (17.7%)	0.426 ^a^
	IVF/ICSI own oocytes	792/4323 (18.3%)	668/3713 (18.0%)	0.362 ^a^
	IVF/ICSI donated oocytes	1059/5782 (18.3%)	762/4369 (17.4%)	0.148 ^a^

^a^ χ^2^ test. * Pregnancy with gestational sac beyond the 12th week and in which no miscarriage had occurred.

**Table 4:** Descriptive analysis of the final pregnancy outcome of all the pregnant women who answered to at least one of the surveys. (1) refers to one live birth and (2) to two live births.

Final Pregnancy Outcome	n	%
Biochemical miscarriage	2	0.2
Clinical miscarriage	39	4.5
Fetal or perinatal death	9	1.0
Live birth (1)	668	76.4
Live birth (2)	21	2.4
Ongoing pregnancy lost to follow-up	135	15.5
TOTAL	874	

**Table 5:** Descriptive analysis of some of the main questions found in the different surveys (first and second trimester, and post-partum).

Variable	Categories	1st Trimester			2nd Trimester			Post-Partum		
		Total	n	%	Total	n	%	Total	n	%
**Hamilton Scale** **Results**	Mild anxiety	439	425	96.8	614	596	97.0	138	132	95.7
	Mild to moderate anxiety		11	2.5		17	2.8		5	3.6
	Moderate to severe anxiety		3	0.7		1	0.2		1	0.7
**Level of Concern** **Regarding COVID-19** **Situation**	Low levels of concern	426	15	3.5	574	9	1.6	-		
	Moderate levels of concern		113	26.5		194	33.8			
	High levels of concern		298	70.0		371	64.6			
**Physical Exercise**	It has been reduced	432	386	89.4	585	339	58.0	-		
	It has remained the same		39	9.0		189	32.3			
	It has been increased		7	1.6		57	9.7			
**Nutrition**	I eat healthier	428	175	40.9	585	273	46.7	-		
	I eat less healthy		30	7.0		21	3.6			
	I eat the same		223	52.1		291	49.7			
**Death of** **Someone Close due** **to COVID-19**	Yes	432	71	16.4	585	85	14.5	-		
	No		361	83.6		500	85.5			
**COVID-19 Test or** **Symptoms**	Positive test	432	1	0.2	585	5	0.9	-		
	Negative test		27	6.3		201	34.3			
	No test but symptoms		14	3.2		11	1.9			
	No test and no symptoms		390	90.3		368	62.9			
